# The 50 most cited studies on posterior tibial slope in joint preserving knee surgery

**DOI:** 10.1186/s40634-022-00557-w

**Published:** 2022-12-12

**Authors:** Romed P. Vieider, Daniel P. Berthold, Armin Runer, Philipp W. Winkler, Phillip Schulz, Marco-Christopher Rupp, Sebastian Siebenlist, Lukas N. Muench

**Affiliations:** 1 Department of Sports Orthopaedics, Klinikum Rechts Der Isar, Technichal University of Munich, Ismaninger Str. 22, 81675 Munich, Germany; 2 Medical University of Vienna, Spitalgasse 23, Vienna, Austria; 3 Department of Orthopaedics and Traumatology, Kepler University Hospital GmbH, Linz, Austria

**Keywords:** Posterior tibial slope, Sagittal alignment, Methodological quality, Osteotomy, Bibliographic analysis, PTS measurement techniques, PTS biomechanics

## Abstract

**Purpose:**

To determine the 50 most cited studies on posterior tibial slope (PTS) in joint preserving knee surgery and assess their level of evidence, objective study quality scores as well as to examine whether the study quality correlated with the citation count and citation density in the top 50 list.

**Methods:**

A literature search on Web of Science was performed to determine the 50 most cited studies on the topic of PTS in joint preserving knee surgery between 1990 and 2022. The studies were evaluated for their bibliographic parameters, level of evidence rating (LOE), citation counts, the Modified Coleman Methodological Score (MCMS), the Methodological Index for Non-Randomized Studies (MINORS), and the Radiologic Methodology and Quality Scale (MQCSRE).

**Results:**

Of the top 50 list, 16 studies were published in the *American Journal of Sports Medicine. A total of 23* studies were produced in the United States (46%). Of 10 different study types, case control studies (*n* = 16, 32%) and cadaveric studies (*n* = 10, 20%) were most common. 15 studies (30%) were purely radiological studies. 6 studies were level II (12%), 23 level III (46%), 15 level IV (30%), and 6 level V studies (12%), respectively. The number of citations ranged from 42 to 447 (mean 105.6 ± 79.2 citations) and showed a mean citation density of 10.3 ± 5.2, composed of the decades 1994 – 2000 (8.3 ± 4.1), 2001 – 2010 (11.1 ± 5.9), 2011 – 2019 (10.1 ± 5.1). Mean quality scores were 55.9 ± 13.0 for MCMS (*n* = 18), 14.5 ± 3.2 for MINORS (*n* = 18) and 18.1 ± 3.7 for MQCSRE (*n* = 20), respectively. High citation counts did not correlate with higher study quality scores (*p* > 0.05). Radiological studies were not significantly cited more often than non-radiological studies (mean 116.9 ± 88.3 vs. 100.8 ± 75.8 citations; *p* > 0.05).

**Conclusion:**

In joint preserving knee surgery, the 50 most cited studies on PTS did not represent a ranking of the highest methodological quality scores. Citation counts and citation density over the past three decades did not significantly differ, even though the number of articles in the presented list multiplied over the same period. This list can serve as a reference tool for orthopedic surgeons aiming to review PTS literature.

## Introduction

Studies describing the morphological characteristics of the knee joint have increasingly been published in recent years [7, 24, 26]. Especially, the posterior tibial slope (PTS), which is defined as the posterior inclination of the tibial plateau in relation to the mechanical axis of the tibia in the sagittal plane, has intensively been investigated [25]. Since studies have outlined the PTS to be a potential independent risk factor for ligamentous, meniscal, and cartilage injuries, PTS gained increasing interest in the orthopedic sports medicine community [17, 43, 48, 49, 55]. More than 1000 articles pertaining to this topic were published since 1978 on NCBIs Pubmed, half of which have been published in the last 3.5 years.

Based on the unfavorable biomechanical effects of increased slope values on the ACL, menisci, and knee cartilage innovation in surgical techniques for treating pathologic PTS values have evolved. Outcomes of surgical limb alignment procedures such as PTS reducing osteotomies show favorable outcomes under ongoing evaluation [2, 15]. Consequently, various imaging modalities have been established to measure PTS in 2D [16, 22] and 3D [28, 34, 38, 41] to determine PTS in clinical diagnostics or accurate preoperative planning. The dissimilar results of various measurement methods, drive an ongoing debate on how to identify the most accurate PTS angle [22, 32, 38] as well as at which cut-off the PTS should be surgically addressed using slope reducing osteotomies [15, 39].

These inconsistencies, combined with the increasing number of publications on PTS, make it difficult to develop a comprehensive foundation on this topic. Review articles that evaluate the most cited references of a particular research field are a common and useful tool to obtain an initial overview about the most impactful publications in almost any medical specialty [3, 6, 9, 11, 18, 19, 35, 36, 52]. Citation counts and the citation indices derived from them are helpful in assessing the impact of journals, authors, or articles and therefore, are frequently used to underline the relevance of literature [12, 21, 47]. Bibliographic characteristics of these studies help the reader to identify the state of knowledge, trends, and centers of competence in a specific field of research, but cannot provide insight into the methodological quality of the study. The increasing quantity of studies in this field make it difficult to assess their quality and establish a reference or place studies in the scientific context. To address this problem, quality scores can give a rough overview of the methodological quality of the studies.

The purpose of the present study was 1) to identify the 50 most cited studies on the topic of PTS in joint preserving knee surgery and their bibliographic parameters and 2) to determine whether the study quality correlated with the citation count and citation density in the top 50 most cited studies.

## Methods

### Literature search

A search of Thompson and Reuters Web of science (WOS) database was performed by R.V. in April 2022. WOS states to list more than 17.1 million records in 254 disciplines (Status April 22). All articles published between 1994 and April the 30^th^ 2022 with available abstracts were included. The search term “posterior tibial slope” (PTS) was used in the searching category “topic” to identify all articles that address this subject. Subsequently, results were filtered by number of citations and ranked with descending order. The complete record of the first 920 studies and related characteristics such as, author name, publication year, journal, title, abstract, total citations (all databases) and language was exported into a data sheet. A comparison of search results with Elseviers Scopus database showed an almost perfect concordance for the term PTS so an additional extraction was not performed. All analysis of the articles was performed in May 2022.

### Inclusion and exclusion criteria

The following inclusion criteria were applied to the exported 920 studies. English articles published in international peer reviewed journals between 1991 and 2022, with the term posterior tibial slope or PTS in their title were included. In addition, the abstracts of all articles were screened to identify studies that did not have the term posterior tibial slope in the title but whose main topic or outcome addressed PTS. Subsequently, these studies were also included.

**Fig. 1:**
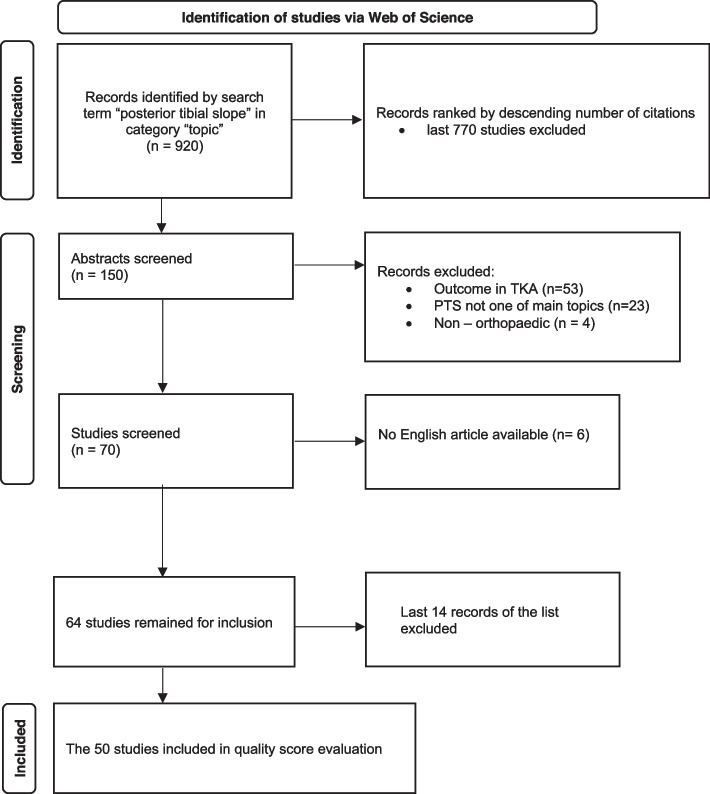
PRISMA flowchart demonstrating the article selection process. Posterior Tibial slope (PTS), Total Knee Arthroplasty (TKA)

Articles that were not published in the field of medicine, whose main topic did not relate to PTS or were not written in English were excluded. Because this study focuses on the field of ligamentous surgery, all articles primarily concerned with outcomes of TKA were also excluded (Fig. 1)*.*

### Data assessment

The included studies were all assessed by R.V. and P.S. for first author, Journal, Journal impact factor of 2021 (JIF), total citation count, study type, country of origin and year of publication. Citation density (total citations divided by years since publication) was further assessed to make older studies, which could tend to have more citations, comparable to recently published articles.

All 50 articles were evaluated for quality using the level of evidence rating (LOE) according to the Journal of Bone and Joint Surgery [40], the Modified Coleman methodological Score (MCMS) [14], the Methodological Index for Non Randomized Studies (MINORS) [44] and the Radiologic Methodology and Quality Scale (MQCSRE) [5]. The MCSM is a reliable tool to evaluate clinical studies and focus on the population size, follow-up rate, diagnostic work up and rehab protocols [14]. The MINORS Score was originally designed for quantifying the methodological quality of observational studies predominantly used in surgical fields [44]. Since the determination of PTS is predominantly performed by radiological methods, several studies focus on determining the best measurement technique or the comparison of different techniques. Therefore, these studies underwent a scale of methodological quality for clinical studies of radiologic examinations (MQCSRE) [5]. No score was obtained for cadaver studies, reviews and meta-analysis or study designs for which the scores were not developed.

### Statistical analysis

Descriptive statistics were calculated using SPSS (IBM, Version 27) and displayed by graphs and tables. Kolmogorov Smirnov test was used to assess if data was normally distributed. Since none of the variables were normally distributed the Mann Whitney U Test or, in case more than two groups were compared, Kruskal Wallis Test was applied for the comparison of citation counts between the different journals. A value of *p* < 0.05 was defined as statistically significant. Two raters (R.V. and P.S.) independently determined the three scores (MCMS, MINORS and MQCSRE) and agreement between them was calculated using Pearson correlation coefficient and cohens kappa (κ). For measurement agreement the following scale was applied: 0–0.2, slight; 0.21–0.4, fair; 0.41–0.6, moderate; 0.61–0.8, substantial; 0.81–1, almost perfect [4].

## Results

### Descriptive statistics

**Fig. 2:**
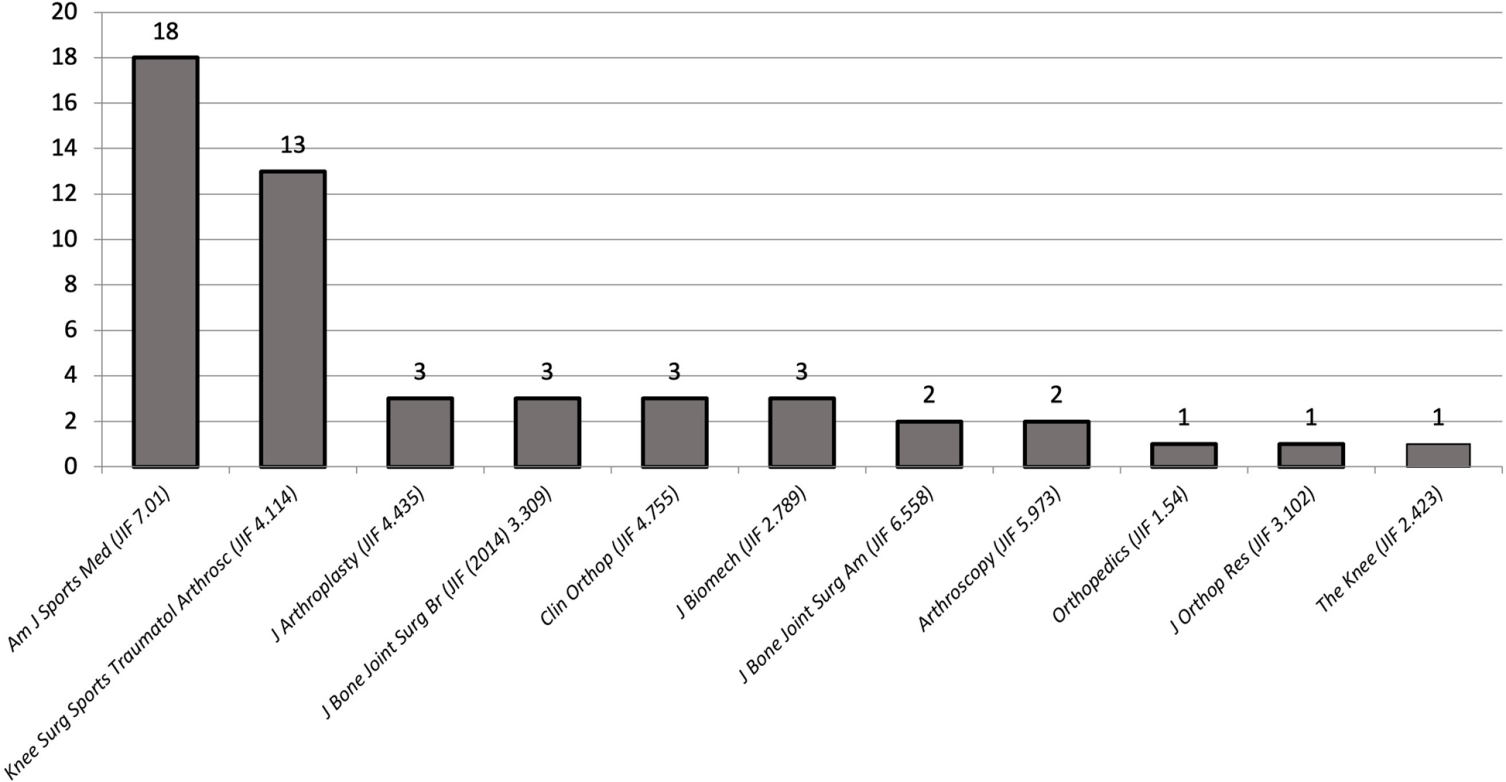
Distribution of the top 50 studies by the journals of publication and their Journal Impact Factor of 2021 (JIF). American Journal of Sports Medicine (Am J Sports Med), Knee Surgery, Sports Traumatology, Arthroscopy (Knee Surg Sports Traumatol Arthrosc), Journal of Arthroplasty (J Arthroplasty), Journal of Bone and Joint Surgery, American Volume (J Bone Joint Surg Am), Journal of Bone and Joint Surgery, British Volume (J Bone Joint Surg Br), Clinical Orthopaedics and Related Research (Clin Orthop), Journal of Biomechanics (J Biomech), Journal of Orthopedic Research (J Orthop Res)

The 50 most cited studies concerning the PTS were cited 5282 times with a mean of 105.6 ± 79.2 citations per article. The articles were published in 10 different journals. “The American Journal of Sports Medicine” (Am J Sports Med), which is listed with a JIF in 2021 (JIF) of 7.01, ranks first with a total of *n* = 18 (36%) of the top 50 list. The second most articles (*n* = 13; 26%) were published by the journal “Knee Surgery, Sports Traumatology, Arthroscopy” (Knee Surg Sports Traumatol Arthrosc) with a JIF 4.114. Both journals combined represented 62% of all included articles published (Fig. 2). The average JIF in the top 50 list was 5.1 ± 1.64, ranging from 7.01 to 1.54. Of all articles, 35.4% were published in journals with a JIF of 5.28 or higher.

**Fig. 3:**
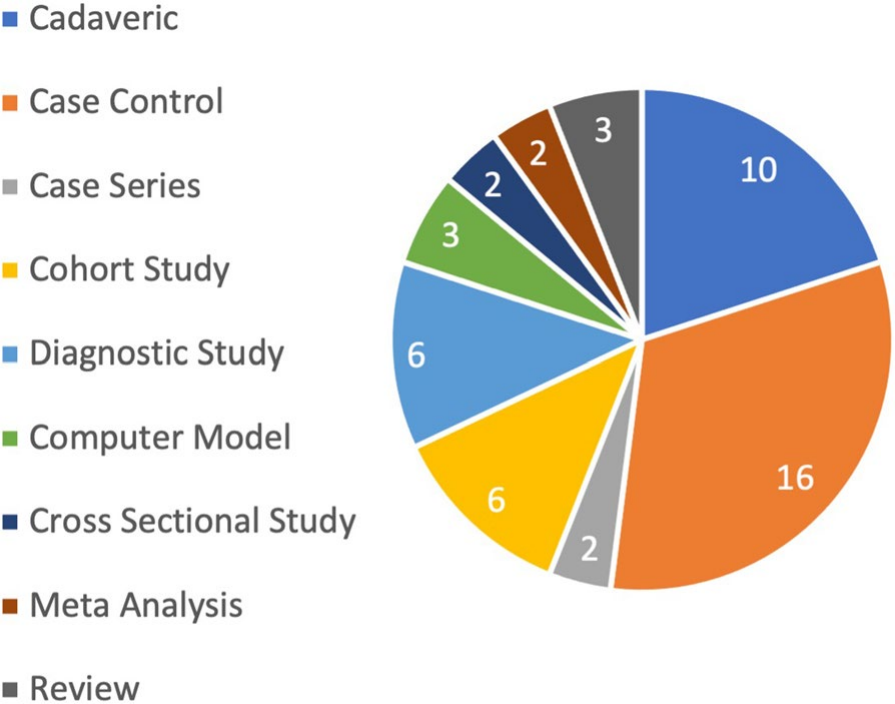
The top 50 studies according to study type

From a total of 10 different study types, case control studies (*n* = 16, 32%) and cadaveric studies (*n* = 10, 20%) were most common (Fig. 3). Fifteen studies (30%) were purely radiological studies. In terms of LOE, 6 Studies were specified as level II (12%), 23 level III (46%), 15 level IV (30%), and 6 level V (12%) studies, respectively.

**Fig. 4:**
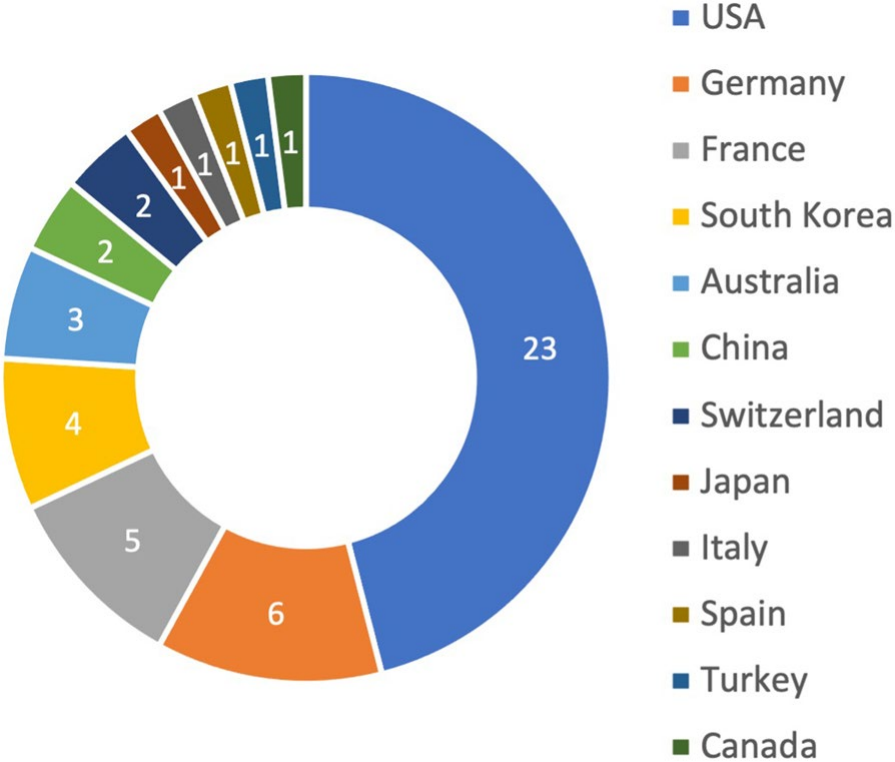
The top 50 studies according to the country of origin

In total, the top 50 articles were published by institutions from 12 different countries with the most articles produced in the United States (US) (*n* = 23; 46%), followed by Germany (*n* = 6; 12%) and France (*n* = 5; 10%) (Fig. 4).

### Quality assessment

Of the top 50 studies, 9 cadaveric studies, 3 reviews, 3 computer model studies, 3 meta-analyses, 1 comparative diagnostic study, 1 cohort study and 1 cross sectional study were excluded from the quality analysis (21 total exclusions) as seen in the comprehensive list (Table 1). The majority of analyzed studies showed a LOE of III (*n* = 23, 46%). Average study quality scores were 55.94 for MCMS (*n* = 18), 14.5 for MINORS (n = 18) and 18.1 for MQCSRE (*n* = 20), respectively. In total, 9 studies (50%) had a MINORS score of 16 or higher, which has widely been regarded as the cutoff for a high-quality study [45].

**Table 1:** Comprehensive list of the 50 most cited studies on posterior tibial slope

Rank	First Author (Year), Journal, Country, *Title*	Citations	Study type	LOE	MINORS	MCMS	MQCSRE
1	**Giffin (2004), Am J Sports Med, US**	**447**	**Cadaveric**	**5**	**N/A**	**N/A**	**N/A**
	*Effects of increasing tibial slope on the biomechanics of the knee*						
2	**Dejour (1994), J Bone Joint Surg Br, FR**	**361**	**Diagnostic Study**	**2**	**N/A**	**N/A**	**20**
	*Tibial translation after anterior cruciate ligament rupture – 2 radiological tests compared*						
3	**Hashemi (2008); J Bone Joint Surg Am, US**	**257**	**Diagnostic Study**		**N/A**	**N/A**	**19**
	*The Geometry of the Tibial Plateau and Its Influence on the Biomechanics of the Tibiofemoral Joint*						
4	**Hashemi (2010), Am J Sports Med, US**	**229**	**Case Control**	**3**	**14**	**62**	**14**
	*Shallow Medial Tibial Plateau and Steep Medial and Lateral Tibial Slopes New Risk Factors for Anterior Cruciate Ligament Injuries*						
5	**Brandon (2006), Arthrocopy, US**	**201**	**Case Control**	**3**	**13**	**N/A**	**N/A**
	*The association between posterior-inferior tibial slope and anterior cruciate ligament insufficiency*						
6	**Simon (2010), J Biomech, US**	**163**	**Case Control**	**3**	**16**	**41**	**13**
	*A case–control study of anterior cruciate ligament volume, tibial plateau slopes and intercondylar notch dimensions in ACL-injured knees*						
7	**Webb (2013), Am J Sports Med, AUS**	**159**	**Case Control**	**3**	**18**	**65**	**N/A**
	*Posterior Tibial Slope and Further Anterior Cruciate Ligament Injuries in the Anterior Cruciate Ligament-Reconstructed Patient*						
8	**Todd (2010), Am J Sports Med, US**	**156**	**Case Control**	**3**	**17**	**56**	**N/A**
	*The Relationship Between Posterior Tibial Slope and Anterior Cruciate Ligament Injuries*						
9	**Hudek (2009), Clin Orthop, SUI**	**152**	**Diagnostic Study**	**4**	**N/A**	**N/A**	**18**
	*Novel Measurement Technique of the Tibial Slope on Conventional MRI*						
10	**Shelburne (2011), J Orthop Res, US**	**147**	**Computer Simulation**	**V**	**N/A**	**N/A**	**N/A**
	*Effect of Posterior Tibial Slope on Knee Biomechanics during Functional Activity*						
11	**Yue (2011), J Arthroplasty, US**	**146**	**Cross sectional**	**4**	**N/A**	**N/A**	**12**
	*Differences of Knee Anthropometry Between Chinese and White Men and Women*						
12	**Giffin (2007), Am J Sports Med, US**	**120**	**Cadaveric**		**N/A**	**N/A**	**N/A**
	*Importance of tibial slope for stability of the posterior cruciate ligament-deficient knee*						
13	**Christensen (2015), Am J Sports Med, US**	**118**	**Case Control**	**3**	**17**	**59**	**15**
	*Lateral Tibial Posterior Slope Is Increased in Patients With Early Graft Failure After Anterior Cruciate Ligament Reconstruction*						
14	**Dejour (2015), Knee Surg Sports Traumatol Arthrosc, FR**	**116**	**Cohort Study**	**3**	**9**	**67**	**N/A**
	*Tibial slope correction combined with second revision ACL produces good knee stability and prevents graft rupture*						
15	**El-Azab (2010), Am J Sports Med, GER**	**106**	**Cohort Study**	**3**			**21**
	*Patellar Height and Posterior Tibial Slope After Open- and Closed-Wedge High Tibial Osteotomy A Radiological Study on 100 Patients*						
16	**Chiu (2000), Am J Sports Med, JAP**	**104**	**Cadaveric**	**4**	**N/A**	**N/A**	**N/A**
	*Posterior slope of tibial plateau in Chinese*						
17	**Sonnery-Cottet (2014), Am J Sports Med, FR**	**103**	**Case Series**	**4**	**10**	**55**	**N/A**
	*Proximal Tibial Anterior Closing Wedge Osteotomy in Repeat Revision of Anterior Cruciate Ligament Reconstruction*						
18	**Yoo (2008), J Arthroplasty, KOR**	**102**	**Diagnostic Study**	**4**	**N/A**	**N/A**	**19**
	*Anatomical references to assess the posterior tibial slope in total knee arthroplasty: A comparison of 5 anatomical axes*						
19	**Beynnon (2014), Am J Sports Med, US**	**97**	**Case Control**	**3**	**16**	**54**	**N/A**
	*Increased Slope of the Lateral Tibial Plateau Subchondral Bone Is Associated With Greater Risk of Noncontact ACL Injury in Females but Not in Males A Prospective Cohort Study With a Nested, Matched Case–Control Analysis*						
20	**Feucht (2013), Knee Surg Sports Traumatol Arthrosc, GER**	**96**	**Review**	**4**	**N/A**	**N/A**	**N/A**
	*The role of the tibial slope in sustaining and treating anterior cruciate ligament injuries*						
21	**Sonnery-Cottet (2011), J Bone Joint Surg Br, FR**	**95**	**Case Control**	**3**	**12**	**38**	**13**
	*The influence of the tibial slope and the size of the intercondylar notch on rupture of the anterior cruciate ligament*						
22	**Wordeman (2012), Am J Sports Med, US**	**95**	**Meta-Analysis**	**3**	**N/A**	**N/A**	**N/A**
	In Vivo* Evidence for Tibial Plateau Slope as a Risk Factor for Anterior Cruciate Ligament Injury A Systematic Review and Meta-analysis*						
23	**Hudek (2011), Clin Orthop SUI**	**94**	**Case Control**	**2**	**15**	**51**	**14**
	*Is Noncontact ACL Injury Associated with the Posterior Tibial and Meniscal Slope?*						
24	**McLean (2011), J Bone Joint Surg Am, US**	**94**	**Cadaveric**	**4**	**N/A**	**N/A**	**N/A**
	*The Relationship Between Anterior Tibial Acceleration, Tibial Slope, and ACL Strain During a Simulated Jump Landing Task*						
25	**El-Azab (2008), J Bone Joint Surg Br, GER**	**94**	**Cohort Study**	**3**	**N/A**	**N/A**	**21**
	*The effect of closed- and open-wedge high tibial osteotomy on tibial slope—A retrospective radiological review of 120 cases*						
26	**Hohmann (2011), Knee Surg Sports Traumatol Arthrosc, AUS**	**91**	**Case Control**	**3**	**16**	**61**	**19**
	*Is there a correlation between posterior tibial slope and non-contact anterior cruciate ligament injuries?*						
27	**Salmon (2018), Am J Sports Med, AUS**	**91**	**Case Control**	**3**	**20**	**90**	**N/A**
	*20-Year Outcomes of Anterior Cruciate Ligament Reconstruction With Hamstring Tendon Autograft The Catastrophic Effect of Age and Posterior Tibial Slope*						
28	**Utzschneider (2011), Knee Surg Sports Traumatol Arthrosc, GER**	**90**	**Cadaveric**	**2**	**N/A**	**N/A**	**22**
	*Development and validation of a new method for the radiologic measurement of the tibial slope*						
29	**Vyas (2011), Knee Surg Sports Traumatol Arthrosc, US**	**63**	**Case Control**	**3**	**16**	**50**	**16**
	*Increased medial tibial slope in teenage pediatric population with open physes and anterior cruciate ligament injuries*						
30	**Song (2007), Orthopedics, KOR**	**61**	**Cohort Study**	**3**	**N/A**	**N/A**	**20**
	*How to avoid unintended increase of posterior slope in navigation-assisted open-wedge high tibial osteotomy*						
31	**Chae (2008), The Knee, KOR**	**60**	**Cohort Study**	**3**	**N/A**	**N/A**	**25**
	*Tibial slope and patellar height after opening wedge high tibia osteotomy using autologous tricortical iliac bone graft*						
32	**Li (2014), Am J Sports Med, CN**	**59**	**Case Control**	**3**	**8**	**55**	**N/A**
	*Posterior Tibial Slope Influences Static Anterior Tibial Translation in Anterior Cruciate Ligament Reconstruction A Minimum 2-Year Follow-up Study*						
33	**Eckhoff (1994), Clin Orthop, US**	**59**	**Cadaveric**	**2**	**N/A**	**N/A**	**N/A**
	*3-dimensional computed tomography reconstruction of tibial torsion*						
34	**Bernhardson (2019), Am J Sports Med, US**	**58**	**Cadaveric**		**N/A**	**N/A**	**N/A**
	*Tibial Slope and Its Effect on Force in Anterior Cruciate Ligament Grafts: Anterior Cruciate Ligament Force Increases Linearly as Posterior Tibial Slope Increases*						
35	**Alentorn-Geli, (2014), Knee Surg Sports Traumatol Arthrosc, ES**	**56**	**Review**	**4**	**N/A**	**N/A**	**N/A**
	*Prevention of anterior cruciate ligament injuries in sports-Part I: Systematic review of risk factors in male athletes*						
36	**Marouane (2014), J Biomech, US**	**55**	**Computer Simulation**		**N/A**	**N/A**	**N/A**
	*Steeper posterior tibial slope markedly increases ACL force in both active gait and passive knee joint under compression*						
37	**Hinterwimmer (2011), Am J Sports Med, GER**	**55**	**Case Series**	**4**	**N/A**	**N/A**	**22**
	*Control of Posterior Tibial Slope and Patellar Height in Open-Wedge Valgus High Tibial Osteotomy*						
38	**Cullu (2005), Knee Surg Sports Traumatol Arthrosc, TUR**	**55**	**Diagnostic Study**	**4**	**N/A**	**N/A**	**22**
	*Tibial slope changes following dome-type high tibial osteotomy*						
39	**Faschingbauer (2014), Knee Surg Sports Traumatol Arthrosc, GER**	**53**	**Diagnostic Study**	**2**	**N/A**	**N/A**	**21**
	*Can the tibial slope be measured on lateral knee radiographs?*						
40	**Voos (2012), Knee Surg Sports Traumatol Arthrosc, US**	**53**	**Cadaveric**		**N/A**	**N/A**	**N/A**
	*Effect of tibial slope on the stability of the anterior cruciate ligament-deficient knee*						
41	**Marouane (2015),J Biomech, US**	**51**	**Computer Simulation**	**N/A**	**N/A**	**N/A**	**N/A**
	*Quantification of the role of tibial posterior slope in knee joint mechanics and ACL force in simulated gait*						
42	**Dargel (2009), Knee Surg Sports Traumatol Arthrosc, GER**	**50**	**Cadaveric**	**4**	**N/A**	**N/A**	**N/A**
	*Side differences in the anatomy of human knee joints*						
43	**Grassi (2019), Am J Sports Med, ITA**	**50**	**Case Control**	**3**	**16**	**43**	**16**
	*Steep Posterior Tibial Slope, Anterior Tibial Subluxation, Deep Posterior Lateral Femoral Condyle, and Meniscal Deficiency Are Common Findings in Multiple Anterior Cruciate Ligament Failures: An MRI Case–Control Study*						
44	**Nunley (2014), J Arthroplasty, US**	**50**	**Cohort Study**	**2**	**N/A**	**N/A**	**N/A**
	*Extreme Variability in Posterior Slope of the Proximal Tibia: Measurements on 2395 CT Scans of Patients Undergoing UKA?*						
45	**Zeng (2016), Knee Surg Sports Traumatol Arthrosc, CN**	**47**	**Case Control**	**3**	**13**	**43**	**N/A**
	*Is posterior tibial slope associated with noncontact anterior cruciate ligament injury?*						
46	**Weinberg (2017), Am J Sports Med, US**	**47**	**Cross sectional**	**3**	**N/A**	**N/A**	**N/A**
	*Differences in Medial and Lateral Posterior Tibial Slope: An Osteological Review of 1090 Tibiae Comparing Age, Sex, and Race*						
47	**Lee (2018), Arthroscopy, KOR**	**46**	**Case Control**	**3**	**15**	**43**	**N/A**
	*Does Posterior Tibial Slope Affect Graft Rupture Following Anterior Cruciate Ligament Reconstruction?*						
48	**Nha (2016), Am J Sports Med, KOR**		**Meta Analysis**	**3**	**N/A**	**N/A**	**N/A**
	*Change in Posterior Tibial Slope After Open-Wedge and Closed-Wedge High Tibial Osteotomy A Meta-analysis*						
49	**Cantin (2015), Knee Surg Sports Traumatol Arthrosc, FR**	**43**	**Review**	**4**	**N/A**	**N/A**	**N/A**
	*The role of high tibial osteotomy in the treatment of knee laxity: a comprehensive review*						
50	**Imhoff FB (2019), Knee Surg Sports Traumatol Arthrosc, US**	**42**	**Cadaveric**	**3**	**N/A**	**N/A**	**N/A**
	*Slope-reducing tibial osteotomy decreases ACL-graft forces and anterior tibial translation under axial load*						

Interrater analyses showed substantial agreement for the MCMS (κ = 0.62) and MINORS (κ = 0.69) and almost perfect agreement for the MQCSRE (κ = 0.866) between the two raters [31].

### Citation counts

Total citation count of all 50 studies was 5282 with a mean of 105.6 ± 79.2 citations per article. Of all citations, 2129 (40.2%) were from articles published in the Am J Sports Med, followed by Knee Surg Sports Traumatol Arthrosc with 855 citations (16.2%) and the Journal of Bone and Joint Surgery (American Volume British Volume combined) with 807 citations (15.3%).

**Fig. 5:**
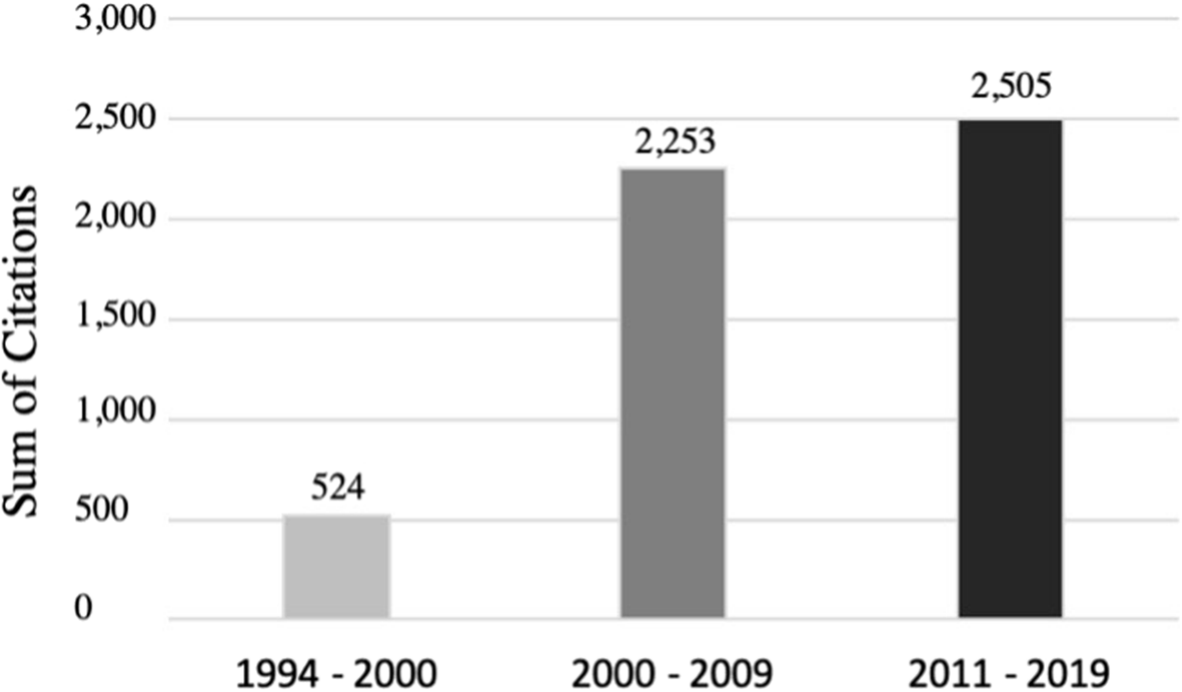
Sum of citations of all articles published according to the last three decades

**Fig. 6:**
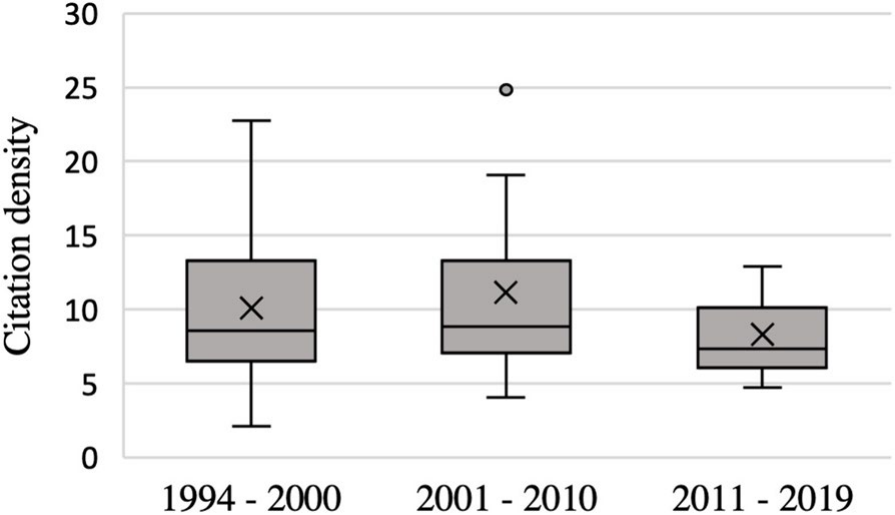
Citation density of all articles published according to the last three decades

When distributing the total citations according to the past three decades, it is apparent that with a total count of 2505, the highest number of citations is between 2011 and 2019 (47.4%) (Fig. 5). However, in this period most articles were published (*n* = 36; 72%). The average number of citations per article in this decade was 78.2 ± 33.3. In contrast, there are a mean of 150.2 ± 104.2 citations per article (*n* = 15) in the period from 2001 to 2010. Citation density in the period between 1994—2000 was 8.3 ± 4.2, between 2001—2010 it was 11.2 ± 5.9, and in the period between 2011 – 2019 mean citation density was 10.1 ± 5.1, respectively (Fig. 6). These results were not statistically significant (*p* > 0.05). The article with the highest citation density (24.8 citations per year) was also the most cited article overall by Giffin et al. [23].

The most citations per article were observed in the “Journal of Bone and Joint Surgery” (American and British Volume combined) and resulted in 201.8 citations per article of total *n* = 4 articles in the top 50 list. Due to the small number in this group, Kruskal Wallis test showed no significant difference of citation count and publishing Journal (*p* > 0.05).

No significant correlation between citation counts and all three quality scores and LOE was found (*p* > 0.05) (Table 2).

**Table 2:** Correlation between citation count vs methodological quality scores and Level of evidence. a Modified Coleman methodological Score (MCMS); Methodological Index for Non-Randomized Studies (MINORS); Radiologic Methodology and Quality Scale (MQCSRE); Level of evidence (LOE)

	Correlation coefficient R	*n* =	*p*-value
Mean citations vs. *MINORS*^*a*^	.105	18	*p* > 0.05
Mean citations vs. LOE^a^	.131	50	*p* > 0.05
Mean citations vs. MQCSRE ^a^	.205	20	*p* > 0.05
Mean citations vs. MCMS^a^	.242	18	*p* > 0.05

**Fig. 7:**
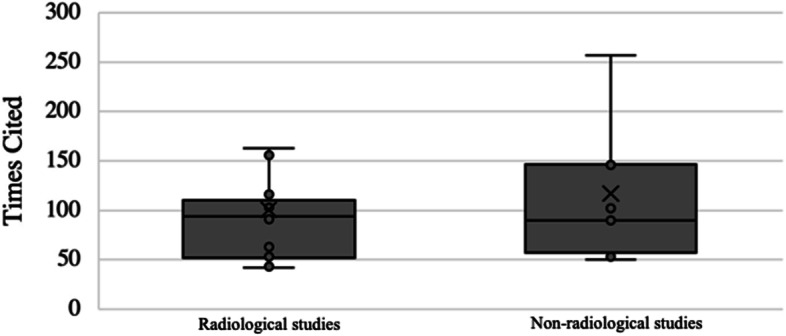
Boxplot diagram and mean citations of radiological and non-radiological studies

A total of 15 purely radiological studies were represented in the top 50 list. While radiological studies tended to be cited more often per article (116.9 ± 88.3 citations/article) than non-radiological studies (100.8 ± 75.8 citations/article) (Fig. 7), this result was not statistically significant (*p* > *0.05*).

There were no significant differences in citation count and country of origin (*p* > *0.05*). Additionally, comparison of mean citations per article according to the four geographical regions of USA (122.5 ± 96.9), Asia (74.4 ± 35.0), Europe (98.4 ± 76.5) or Australia (113.7 ± 39.3) revealed no significant difference (*p* > *0.05*).

## Discussion

The most important finding of this study was, that objective quality scores did not indicate a higher citation count. The overall most cited study by Giffin et al. [23] investigated the biomechanical impact of the PTS on the knee joint. This cadaveric study was published in 2004 and cited 447 times. The citation density of this article showed 24.8 citations per year, which also ranks it first in this category. Since this study was an experimental cadaver study and none of the used quality scores would fit this study design, no score was obtained. The second most cited study was the 361 times cited Dejour and Bonnins’ radiological comparison of two tests in healthy and ACL deficient knees published in 1994. This study shows a citation density of 12.9 citations per year, which may not look like much at first sight, but is a significant number considering that it was published over 27 years ago. It resulted in a MQCSRE of 20, which is the median compared to the other purely radiological studies. Hashemi's two studies about potential tibial risk factors for ACL injuries [26] and interindividual characteristics and prevalence of tibial morphology [26] were ranked 3rd and 4th in the list with 257 and 229 citations, respectively. Interestingly, the study of Hashemi addressing morphological risk factors for ACL injury ranked 4^th^ in the top 50 list but appeared to obtain low quality scores (MCMS: 62, MINORS: 14 MQCSRE: 14).

Overall, the first four studies of the top 50 list represent a rough categorization (Biomechanics, Radiological measurement, prevalence of PTS angles and clinical effects of high PTS values) of the top 50 list. Sixteen studies (32%) were case control studies which represented the most frequently used study design. Eleven cadaveric studies (22%), of which eight were investigations on the biomechanical impact of the PTS on knee joints in a laboratory setting. The third most study design were cross sectional studies to investigate the prevalence of PTS values. Among this study type, the three-dimensional CT and MRI investigation of knee joints in healthy Chinese subjects of Yue et al. was cited 146 times (11^th^ rank overall) [54]. There were no prospective studies in the top 50 list, which was reflected in the LOE rating 44 out of 50 were level III or Level IV studies according to the LOE published by Wright in the JBJS in 2003 [50] and shows the lack of level I and level II evidence in this field of research. Especially, randomized prospective cohort studies would be needed to provide further evidence with the knowledge that this is not always possible with surgical fields.

The topic on posterior tibial slope gained immense popularity over the last 10 years. This shows the impressive result, that in the past three decades, the number of articles multiplied from 3 (1990 – 2000) to 15 studies (2001 – 2010) and 32 studies (2011–2020). Nevertheless, of the top 10 most cited studies, only 3 were published in the last decade and shows the independence of the studies’ citation count in regards of their year of publication. This is underlined by the similar citation density of the articles over the assessed three decades.

There are various causes for an increasing interest in PTS in the orthopedic sports medicine and joint regenerative community. Firstly, surgeons are still struggling with unsatisfying failure rates in ACL reconstruction surgery, which have been reported to be as high as 19.3% of cases [30, 37]. The PTS was identified as a singular risk factor not only for graft failure but also in primary ACL injury [7, 13, 26]. Biomechanical studies could emphasize similar results, that the increase of PTS is resulting in an increased load on the ACL [8, 25, 33]. A long term 20 year clinical follow up study by Salmon et al. showed that clinical findings correlated to the biomechanical findings [42]. The authors reported a graft survival rate of 22% with PTS values more than 12 degrees [42]. Salmons’ study has already been cited 91 times to date. In this study true lateral X-rays of the tibia were used to assess the PTS.

However, X-rays are only one imaging modality to determine the PTS. Different measurement methods have been evolved so it is sometimes hard to interpret slope values of different patient cohorts. Four studies of the top 50 list proposed a measurement method to determine the PTS on different imaging modalities [16, 25, 28, 46]. Additionally, Yoo et al. investigated the different anatomical axis of the tibia and the resulting effects on the measured PTS [53]. Utzeschneider et al. compared measured results in long vs. short tibial axis x-rays [46]. All of the mentioned measurement techniques show inherent reliability but their comparability among each other are to a very limited extend [27, 32, 34, 38]. Outside of this list, there are other studies that compare measurement methods, showing that ultimately the literature does not conclusively agree on which method is best [10, 38, 51]. One has to admit, that due to the prompt, cheap and widespread availability of radiography, the PTS is predominantly measured on true lateral radiographs in clinical practice. Although, radiological studies were cited more frequently than non-radiologic studies, there was no underlying statistical significance.

Individual morphological differences among patients, coupled with the different measurement methods and the so far unclarified definitive pathological cutoff value of the PTS, make it difficult to obtain an initial overview of this topic and find the most impactful publications. It is a common method, not only in orthopedic surgery but also in other disciplines, to provide a rough overview of certain issues bibliographic listing of the most cited studies [3, 19, 52]. Furthermore, it is nevertheless important to consider citation counts and methodological quality separately. A study is not of high quality because it is often cited and vice versa as in this study high citation counts did not correlate with high study quality scores, too. This article provides a bibliographic listing of the most cited studies with a quality assessment and their study type at a glance.

Since the PTS is of particular interest in knee reconstructive and sports orthopedic surgery it fits the result, that approximately two out of three studies were published in the Am J Sports Med or Knee Surg Sports Traumatol Arthrosc (*n* = 31, 62%). The aims and scope of these two journals comprise sports related injuries, regenerative joint surgery in athletes and rehabilitation. Out of a total of 5282 citations, 2984 (56.6%) were from these two journals. It is therefore comprehensible that 31 studies address the difference of clinical outcomes in ACL reconstruction or emphasize the PTS as a risk factor of primary anterior cruciate ligament injuries.

The 50 articles were published out of twelve different countries. The dominant proportion of US studies (*n* = 23) is consistent with similar bibliometric studies and is not unique to orthopedic specialties [1, 3, 19, 35, 36]. This could result in authors outside the US being read and cited less than US authors. European institutions published 16 studies, with Germany (*n* = 6) and France (*n* = 5) leading the way. It reflects the importance of European institutions on this topic.

This study has several limitations. Firstly, quality assessment of studies is a very important tool to make them comparable and to objectivize their design and methodology. A randomized controlled trial (RCT) that is triple-blinded is considered the neatest type of interventional or clinical studies. Especially, in surgical specialties RCTs are not always implementable in daily clinical routine. Every score used in this study, was originally designed for a certain study type. The subjectivity of single scores could bias the grading of some articles. The MCMS Score was originally designed to compare the methodological quality of cohort studies and favors prospective cohort studies [14]. Most of the studies in this top 50 list are case control studies or retrospective cohort studies and therefore, are graded up to 15 points less due to their study type. The MINORS Score interpretation favors studies with a long follow up period and a precise rehab protocol [44]. 15 studies were purely radiologic studies; thus a radiological quality score was determined. Consequently, comparability between the different scores is limited. It was not possible to determine a quality score for every study. Furthermore, there were a total of 21 studies for which no quality scores were calculated due to their study design. There are some effects resulting from the dynamics and mechanisms of scientific writing. Researchers tend to use highly cited articles more often in their manuscripts. This creates a snowball effect that could distort the results [29]. It is difficult to numerically capture and objectively estimate this effect. Furthermore, studies which were published earlier are averagely cited more than recently published work, which showed this article too. Citation density is a simple tool to put absolute citation counts into context of time. In contrast to this, the so-called obliteration of incorporation effect can underestimate citation counts. This effect describes the incorporation of widespread accepted knowledge into recent publications and results in a lesser citation count of older studies in particular [20].

## Conclusion

Higher citation counts did not correlate with higher objective methodological quality scores in the 50 most cited articles on PTS. Citation counts and citation density over the past three decades did not significantly differ, even though the number of articles in the presented top 50 list multiplied over the same period.

## Abbreviations

ACL: Anterior Cruciate Ligament
Am J Sports Med: American Journal of Sports Medicine
J Bone Joint Surg Am: Journal of Bone and Joint Surgery American Volume
J Bone Joint Surg Br: Journal of Bone and Joint Surgery British Volume
Knee Surg Sports Traumatol Arthrosc: Knee Surgery, Sports Traumatology, Arthroscopy
LOE: Level of Evidence
MCMS: Modified Coleman methodological Score
MINORS: Methodological Index for Non-Randomized Studies
MQCSRE: Methodological Quality For Clinical Studies Of Radiologic Examinations
PTS: Posterior Tibial Slope
TKA: Total Knee Arthroplasty
WOS: Web of Science
US: United States
